# American Medical Biography; or, Memoirs of Eminent Physicians, Embracing Principally Those Who Have Died Since the Publication of Dr. Thacher’s Work on the Same Subject

**Published:** 1845-02

**Authors:** 


*American Medical Biography; or, Memoirs of Eminent Physicians, embracing principally those who have died since the publication of Dr. Thacher's work on the same subject. With engravings.* By Stephen W. Williams, M. D., Late Professor and Lecturer upon Materia Medica, Medical Jurisprudence, etc., in the Medical College of Berkshire, Mass., etc. etc. 8vo. pp. 664. Greenfield, Mass.: 1844.

This work is dedicated by the author, with much propriety, "to the medical profession throughout the United States;" and we hope that its ready and extensive sale will prove that it is, as it ought to be, an acceptable offering.

"Fame," it has been said, "is the recompense of the dead, not of the living," and there is truth as well as poetry in the expression: but the living may well profit by the example afforded in a review of the actions of the dead, for which fame is the recompense. What is experience but the history of the past? And what history can guide us so well as the history of those whose objects and pursuits were identical with our own? Biography we have ever regarded as the most interesting branch of history; and if the lives of eminent physicians were more generally written and read, the world would be the wiser and the better. If every young physician, especially, were to make himself familiar with the life and sentiments of his eminent predecessors, we are persuaded that it would tend very greatly to elevate the general character of the profession. Entertaining these views, we are always pleased when we meet with a frank and unostentatious account of the lives of departed physicians, who have toiled long and honourably in the profession. In Dr. Williams' work, we discover nothing that does not seem to us perfectly fair and candid—no effort to exalt one at another's expense—certainly no disposition to detract from the fair fame of any one of whom he has written. For the most part his biographical notices are supplied by the immediate friends of the deceased, or drawn up from materials furnished by them. So far from any attempt to exclude the worthy, if we were inclined to find fault with the author, we might say that he has been unnecessarily liberal. We have been surprised, indeed, to find so many names in the book presented for the first time to our notice, particularly from Massachusetts and the adjoining states. This preponderance of eastern names has proceeded, it is probable, from the ready access to materials afforded the author in the well kept records of the Boston Medical and Surgical Journal.

Among the more prominent of those who are commemorated in this volume, we find the names of Miller, Hosack, Mitchell, Miner, Post, Macneven, James, Dewees, Physick, Parrish, Eberle, Nathan Smith, and Godman.

The work is handsomely printed, with good type, white paper, lithographed likenesses, and beautifully bound.

